# Objectively Assessed Weight Change and All-Cause Mortality among Community-Dwelling Older People

**DOI:** 10.3390/nu14142983

**Published:** 2022-07-21

**Authors:** Tagrid Alharbi, Joanne Ryan, Rosanne Freak-Poli, Danijela Gasevic, Jacqueline Scali, Karen Ritchie, Marie-Laure Ancelin, Alice J. Owen

**Affiliations:** 1 School of Public Health and Preventive Medicine, Monash University, 553 St. Kilda Rd, Melbourne, VIC 3004, Australia; tagrid.alharbi@monash.edu (T.A.); joanne.ryan@monash.edu (J.R.); rosanne.freak-poli@monash.edu (R.F.-P.); danijela.gasevic@monash.edu (D.G.); 2 Department of Epidemiology, Erasmus Medical Centre, 3015 GD Rotterdam, The Netherlands; 3 Usher Institute, University of Edinburgh, Teviot Place, Edinburgh EH8 9AG, UK; 4 INM, Université de Montpellier, INSERM, 34000 Montpellier, France; jacqueline.scali@inserm.fr (J.S.); karen.ritchie@inserm.fr (K.R.); marie-laure.ancelin@inserm.fr (M.-L.A.); 5 Centre for Clinical Brain Sciences, University of Edinburgh, Edinburgh EH16 4SB, UK

**Keywords:** weight change, weight loss, weight gain, body weight, body weight maintenance, healthy aging, ageing, aged, older adults, mortality

## Abstract

Later life changes in body weight may be associated with an increased risk of mortality in older adults. The objective of this study was to examine whether weight change over four years was associated with a 17-year mortality risk in older adults. Participants were 1664 community-dwelling adults aged ≥65 years in the longitudinal Enquete de Sante’ Psychologique-Risques, Incidence et Traitement (ESPRIT) study. Outcomes were all-cause mortality, cardiovascular disease (CVD) and cancer mortality. Weight change was defined as difference between weight at baseline and 4 years, categorised into: weight stable (±<5% weight change), weight loss (≥5%) and weight gain (≥5%). Association between weight change and mortality risk was evaluated using Cox proportional hazards models. Over 17 years of follow-up (median 15 years), 565 participants died. Compared to stable weight participants, those with ≥ 5% weight loss had an increased risk of all-cause mortality (HR: 1.24, 95% CI: 1.00–1.56, *p* = 0.05) and CVD mortality (HR: 1.53, 95% CI: 1.10–2.14, *p* = 0.01), but not cancer mortality (HR: 0.83, 95% CI: 0.50–1.39, *p* = 0.49). Weight gain of ≥5% was not associated with increased mortality (HR: 1.05, 95% CI: 0.76–1.45, *p* = 0.74). Weight monitoring in older adults could help identify weight loss at its early stages to better target interventions to maintain nutritional reserve and prevent premature mortality.

## 1. Introduction

The number of adults aged 65 years and over is increasing rapidly, with expectations that it will grow from 9.3 % in 2020 to 16.0 % by 2050 [1]. Following the trend of a global aging population, the prevalence of obesity in the older population is continuing to increase [2,3,4]. Obesity is an established risk factor for metabolic and cardiovascular diseases and certain types of cancer [5], and may also exacerbate functional decline in older age [6]. Therefore, maintaining health and independence in older adults presents a challenge considering the susceptibility of this age group to chronic diseases and functional decline.

Body composition tends to change as people age; fat mass increases, muscle mass decreases, bone mass decreases and body fat distribution shifts [7]. These changes that occur in later life can influence physical function and are often hypothesised to be an indicator of more complex health problems or undiagnosed underlying diseases. Studies have demonstrated that overweight and obesity in middle age is a strong risk factor for cardiovascular disease (CVD) and mortality [8,9]; however, the nature of the relationship between overweight/obesity and mortality appears to change with age [10,11]. There is evidence to suggest U-shaped and reverse J-shaped relationships between body mass index (BMI) and mortality risk among older adults [9,12,13].

Many previous cohort studies have assessed the association between BMI and mortality by examining BMI or weight at only a single point in time [14,15,16]. This ignores the likely changes in weight over time. The association between weight change and health outcomes may also differ between younger and older adults. The mechanisms by which weight loss in older life increases mortality risk may be mediated by a variety of pathways. For example, decreases in muscle mass or/and muscle strength (sarcopenia) and loss of bone mass (osteopenia or osteoporosis) may be associated with an increased risk of morbidity and mortality in older adults [17,18]. Sarcopenia can also occur in the presence of obesity, often described as sarcopenic obesity [19].

Considering the shifts in body composition distribution in older age, weight change may be a better mortality predictor than a single weight assessment in older adults [20]. A recent meta-analysis including 30 studies of older adults aged 65 years and over demonstrated that weight loss was associated with a 59% increase in all-cause mortality, while weight gain was associated with a more modest 10% increase in all-cause mortality in the pooled analysis compared to stable weight, and not all studies found weight gain to be associated with increased risk of mortality [21]. Method of weight measurement (e.g., self-reported versus objectively measured) and length of follow-up were major sources of heterogeneity between studies contributing to the meta-analysis. In light of the limited number of studies examining objectively measured weight change and long-term mortality outcomes, this study aimed to determine whether weight change in older adults over a four year time period is associated with all-cause mortality, as well as cause-specific mortality (CVD and cancer).

## 2. Materials and Methods

### 2.1. Study Population

The data are from the French longitudinal ESPRIT (Enquete de Sante’ Psychologique-Risques, Incidence et Traitement) study [22]. The ESPRIT eligibility criteria resulted in recruitment of a cohort who were community-dwelling older adults aged ≥65 years (living independently), French-speaking, residing in the Montpellier district in southern France and were randomly drawn from 15 electoral rolls across the Montpellier district, between March 1999 and February 2001. Ethical approval for the study was granted by the Ethical Committee of University Hospital of Kremlin-Bicetre, France, and written consent was obtained from each participant. Further details of the ESPRIT study have been reported elsewhere [22,23].

### 2.2. Exposure: Weight Change

Weight was measured in all subjects while lightly dressed in winter or in summer-weight clothing within 1 to 2 h of their arrival during the medical examination at baseline (1999–2000) and at four year follow-up (2003–2004) by trained nurses using the scales available in the clinic. Weight change was calculated as the difference in objectively measured body weight over four years between baseline in 1999–2000 and four-year examinations in 2003–2004. Weight change was categorised into weight loss (≥5%), weight stable (±<5%) and weight gain (≥5%), as a ≥5% weight change from baseline is commonly accepted as being clinically relevant [24].

### 2.3. Outcome: Mortality

At each follow-up, participants were contacted and when they could not be reached, mortality status was ascertained by contacting relatives and general practitioners or the civil registry. The mortality status was ascertained for all participants over a maximum of 17 years of follow-up with a median follow-up time of 15 years. Date and cause of death was determined from death registries and medical records (based on the 10th version of International Classification of Disease (ICD–10)), as detailed previously [25,26]. All-cause mortality was the principal outcome and cause-specific mortality, in particular cancer and cardiovascular disease (CVD) mortality, were also examined. Immediate or underlying cause coded between (I00-I78) or equal to R960 defined CVD mortality, and those between (C00-C97) or between (D00-D48) defined cancer mortality.

### 2.4. Sociodemographic and Clinical Variables

At baseline, a standardised self-completed questionnaire was used to collect demographic characteristics, which included age, sex, marital status (married, divorced/separated, widowed, single), smoking status based on any intake (never smoker, or past/current smoker), alcohol consumption status based on any intake (non/past drinker, drinker), completed secondary school (yes, no). At baseline and then each subsequent follow-up, at a face-to-face interview, participants self-reported information on history of cancer in the past two years, and diabetes (fasting glycaemia 7.0 mmol/L or reported treatment). Additionally, body weight (kg), height (m), and waist circumference (cm) were independently measured at baseline. Body mass index (BMI) was computed as weight (kg) divided by height (m) squared. Current depression was defined as current high depressive symptoms (CESD) or clinically diagnosed major depressive disorder (MDD) [22].

### 2.5. Study Sample

Of the 2270 participants recruited to the study, 606 participants did not have objectively measured weight at both baseline and four years (Supplementary Figure S1). The present analysis was thus conducted on 1664 participants. Compared with the analysed sample, participants excluded from the analysis were more likely to be older (mean 74.9 vs. 72.7 years, *p* < 0.001), have depression (38.9% vs. 28.1%, *p* < 0.001) and diabetes (12.2% vs. 9.0%, *p* = 0.03), but less likely to have completed at least secondary school (26.8% vs. 35.2%, *p* < 0.001), or to be non-alcohol drinkers (21.9% vs. 15.2%, *p* < 0.001). However, there were no significant differences for sex, marital status, smoking status, cancer and baseline BMI between analyses of participants and those excluded from the analysis (*p* > 0.05 for all) (Supplementary Table S1).

### 2.6. Statistical Analysis

Summary statistics were used to describe the frequencies or means (±SD) of the baseline characteristics presented by weight change group. Chi-square or ANOVA tests were applied to compare the distributions of categorical and continuous variables, respectively, across the weight change groups. Cox proportional hazard regression models were used to examine the associations of weight change groups with all-cause mortality, cause-specific mortality (cardiovascular disease and cancer). For cause-specific mortality analysis, a competing-risks model was used with the remaining causes of death as competing events. Hazard ratios (HRs) and 95% confidence intervals (CIs) were reported. Age was used as the time scale, and baseline age was used as the time origin to account for non-proportionality in risk of mortality with age, as detailed previously [27]. The following series of models were constructed: Model 1 was adjusted for sex; Model 2 was additionally adjusted for education, smoking status, alcohol consumption and marital status; Model 3 was as per Model 2 with additional adjustment for diabetes, recent cancer, depression, baseline BMI and waist circumference. Baseline variables were considered as covariates based on those sufficiently common and widely recognised to contribute to the risk of mortality in older individuals, while at the same time theoretically could contribute to weight change, and not be considered to be on a causal pathway between weight change and mortality. This included demographic factor variables and clinical measures variables [21,28]. In addition, a restricted cubic spline model was used to examine the relationship between continuous percentage weight change and all-cause mortality with five knots: 5th, 25th, 50th, 75th and 95th percentiles using Cox proportional hazards models adjusted for Model 3.

Potential effect modification by sex (i.e., sex specific effects of the association between weight change and mortality) was investigated by including an interaction term of sex and weight change into the Cox regression model, and if significant, sex-stratified analyses were undertaken. In addition, power calculations were performed to quantify the power of the analysis to detect the effect sizes that were found assuming a 5% type I error rate, and statistical significance was set at a two-sided *α* of 0.05. Sensitivity analyses were undertaken based on the previous models by firstly examining ±5 kg weight loss and gain instead of a percentage change, as this has been used in some previous studies [29,30,31] and all-cause mortality and cause-specific mortality. Secondly, models were examined excluding participants who were diagnosed with cancer at baseline, to exclude weight loss that could be directly related to recent cancer or treatment. Thirdly, analysis was undertaken excluding participants with a weight change of more than 20% (*n* = 17), to ensure extreme weight loss/gain was not having a major influence on the findings. Finally, a series of sensitivity analyses were conducted to check the robustness of the results which included censoring of deaths in the first year following weight change exposure, using time in the study, instead of age, as the time-scale and models using age at the 4 year follow-up (age at commencement of the risk period) for time scale, instead of age at baseline.

Analyses were performed in Stata statistical software version 16.0 (StataCorp LLC, College Station, TX, USA; www.stata.com). Data were accessed (1 March 2021) (A *p*-value of <0.05 was used to denote statistical significance.

## 3. Results

### 3.1. Characteristic of the Study Population

There were 1664 participants (Table 1) and the median follow-up time was 15 years (IQR: 11.2–15.7). During this time, 565 (33.9%) participants died. Compared to the weight stable group participants with weight loss (*n* = 271, 16.3%) were more likely to be older and have diabetes, and were more likely to have depression. However, in the weight gain group (*n* = 197, 11.8%), a greater proportion of participants were women compared to the weight stable and weight loss groups, and they have a lower baseline weight and were less likely to be alcohol drinkers or to have depressive symptoms. Marital status, education, smoking status, waist circumference and presence of recent cancer did not differ significantly among weight change groups (Table 1).

### 3.2. Association between Weight Change and All-Cause Mortality

The results of the Cox proportional hazards model indicated that weight loss, in comparison to stable weight, was associated with an increased risk of mortality (HR: 1.27, 95% CI: 1.03–1.56, *p* = 0.02) when adjusting for age and sex (Model 1), and this decreased slightly to an increased risk (HR: 1.24, 95% CI: 1.00–1.56, *p* = 0.05) after further adjustment for any of the adjustment schemes (Model 3; Table 2). In contrast, compared to the weight stable group, weight gain was not associated with all-cause mortality in either the minimally-adjusted or fully-adjusted multivariate models (Model 1–3; Table 2). As the interaction between sex and weight change was not significant (*p* = 0.92), sex-stratified analyses were not undertaken. This analysis had 80% power to detect a HR of 1.30 for weight loss and all-cause mortality, and 83% power to detect a HR of 1.32 for weight gain and all-cause mortality.

**Figure 1:**
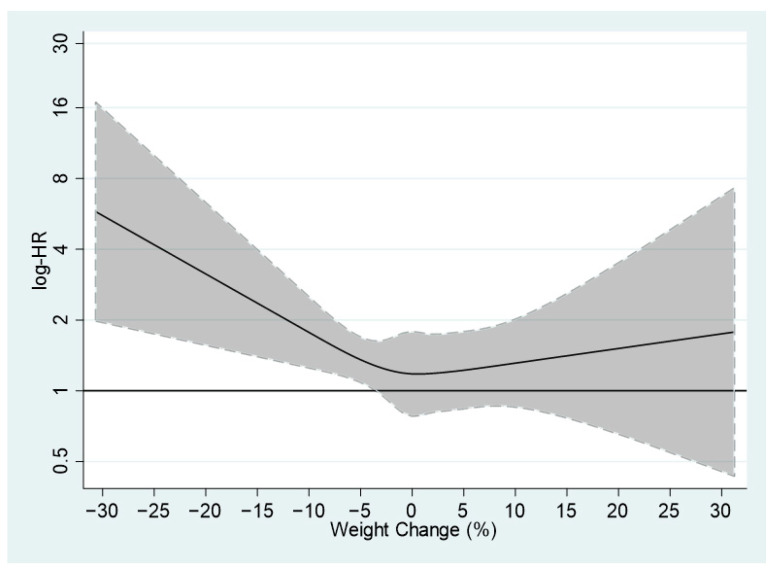
The risk of all-cause mortality according to percentage weight change, estimated by multivariate Cox regression based on restricted cubic splines. Log hazard ratios (log-HRs) with 95% confidence intervals for all-cause mortality and weight change in percentage. The model includes age as time scale, sex, marital status, education level, smoking status, alcohol consumption, depression, diabetes and baseline BMI. Subjects with over than 30 % weight change were not included in plot (*n* = 2).

Investigating the association further between weight change percentage (as a continuous variable) and all-cause mortality risk multivariate adjusted Cox regression with restricted cubic splines was used (Figure 1). The results indicated a positive linear relationship between weight loss percentage and the risk of all-cause mortality; however, weight gain percentage was not associated with mortality risk.

### 3.3. Association between Weight Change with Cause-Specific Mortality and Competing Risks

Compared to participants who were weight stable, weight loss was associated with increased CVD mortality even after multivariate adjustment (weight loss: 64 deaths) (HR: 1.53, 95% CI: 1.10–2.14, *p* = 0.01) (Model 1–3; Table 3) and for other causes (HR: 1.58, 95% CI: 1.17–2.12, *p* = 0.003) (Supplementary Table S6). On the other hand, weight gain was not significantly associated with CVD mortality risk (weight gain: 24 deaths) and other causes. This analysis had 88% power to detect an HR of 1.6 for weight gain and CVD mortality. Overall, the association between weight change (loss or gain) with cancer mortality and other causes was not statistically significant compared to weight stable (weight loss: 22 cancer deaths and weight gain: 18 cancer deaths) (Model 1–3; Table 4) (Supplementary Table S7). This analysis had 86% power to detect an HR of 1.7 for weight loss and cancer mortality. In addition, this analysis had 84% power to detect an HR of 1.7 for weight gain and cancer mortality.

### 3.4. Sensitivity Analysis

When examining the association between ±5 kg weight change (instead of % weight change) and all-cause mortality risk, weight loss of more than 5 kg was significantly associated with higher risk of all-cause mortality (Model 3; HR: 1.70, 95% CI: 1.35–2.18, *p* < 0.001) compared to the weight stable group (weight ± 5 kg over four years). A weight gain of ≥5 kg was not significantly associated with all-cause mortality (Model 1–3; HR: 1.10, 95% CI: 0.74–1.64, *p* = 0.62) (Supplementary Table S2). In addition, ±5 kg weight change and cause specific mortality (CVD and cancer) were examined, the result did not differ markedly. (Supplementary Tables S3 and S4).

In the analyses excluding the 42 participants who reported a recent cancer at baseline, the results remained consistent (Supplementary Table S5), with an increase in significance and effect size for the association between weight loss group and all-cause mortality risk compared to those who were weight stable (HR: 1.30, 95% CI: 1.04–1.63, *p* = 0.02). In the further sensitivity analysis excluding 17 participants with a weight change of more than 20%, the effect sizes remained consistent, although they became non-significant in Model 3 (HR: 1.20, 95% CI: 0.96–1.52, *p* = 0.11) (Supplementary Table S6). Excluding the deaths within the first year of the at-risk period, findings were similar and the results of these analyses are not shown. Moreover, the results of various sensitivity analyses (Supplementary Tables S9–S10) remained consistent with the main findings presented. When the time in the study was used instead of age as the time scale, the effect size for weight loss increased slightly from HR 1.24 to 1.28. However, when age at 4 years follow-up was used to generate the time scale, the effect size for weight loss decreased slightly from HR 1.24 to 1.20.

## 4. Discussion

In this study of community-dwelling older adults aged ≥65 years, objectively measured weight loss over four years was associated with an increase in all-cause mortality risk over a 17-year follow-up. Weight loss, compared to a stable weight, was also associated with increased CVD mortality. No association was observed between weight change and cancer-specific mortality. Interestingly, objectively measured weight gain (≥5%) was not associated with an increased risk of all-cause mortality or cause-specific mortality. Findings were consistent when weight change in kilograms was examined, instead of percentage weight change, and in sensitivity analysis excluding individuals reporting the presence of recent cancer at baseline. Overall, the findings support a non-linear or a reverse J-shaped relation between weight change and all-cause mortality.

The present findings are consistent with the results of previous meta-analyses on the association between weight loss and mortality risk [21,28,32]. For example, a recent systematic review and meta-analysis reported that weight loss was associated with a 59% increased mortality risk in adults aged 65 years or over [21]. Results of another meta-analysis indicated a 67% increase in all-cause mortality risk associated with weight loss among adults aged 60 years or over [28]. In addition, the results of a meta-analysis on change in body weight and mortality among those aged 40 to 65 years indicated that weight loss was associated with a 45% increase in all-cause mortality risk, a 50% increase in CVD mortality, and a 19% increase in cancer mortality [32].

There are a number of mechanisms by which weight loss in older adults may increase mortality risk, and these include development of frailty associated with sarcopenia and loss of bone mass [17,18]. Another possible explanation is reverse causality, as weight loss in older adults can be indicative of underlying disease. To attempt to address or explore the issue of reverse causality, subjects with previous cancer at baseline were excluded and observed that the mortality risk associated with weight loss was maintained. However, after the excluded participants (*n* = 17), with more than 20% of weight change, the association became non-significant after multivariate adjustment, probably due to insufficient power arising from decreased numbers of events.

In the present study, weight gain was not significantly associated with all-cause mortality risk nor CVD or cancer-specific mortality. This contrasts the findings of meta-analyses, which have reported a modest but significant associations between weight gain and all-cause mortality [21,28,32]. This could possibly be impacted by the obesity paradox, supported by epidemiological studies in older adults that overweight and obesity may not affect all-cause or specific cause of mortality [33]. Additionally, the concept of having a ‘nutritional reserve’ through overweight or obesity might be protective against malnutrition in older age. Furthermore, being overweight or obese may provide a biological benefit as access to weight provides anti-inflammatory immune mediators [34] and prevention of muscle wasting and weakness (frailty) which may increase resistance to falls [35]. However, the previously reported associations between weight gain and mortality in older adults have been conflicting.

Some studies have found that a 5% weight gain is not associated with increased mortality risk [30,31,36,37]. In agreement with the findings, a study among 4714 community-dwelling Americans aged ≥ 65 years reported that 5% weight gain after three years was not associated with mortality risk over four years. On the other hands, other studies have reported that a 5% weight gain was associated with mortality risk in older adults [29,38,39], with some of these examining weight change based on recalled weight. Park et al. [29] reported an increased risk of mortality with large weight gain after a period of 10 years of weight change (>10 kg; HR of 1.41, 95% CI 1.13–1.76) but not for small weight gain (>5 kg; HR of 0.98, 95% CI 0.84–1.14) during an average of 7.3 years of mortality follow-up in adults aged 65–75 years. In a study of Korean adults aged 65 or over, weight gain of 5% over a period of four years was associated with a 10% increase in mortality, although that study had a relatively short follow-up period of five years [38]. Another study among 882 Swedes aged 70–97 years reported [39] that a 5% BMI gain over two years was associated with 53% increased mortality risk over 18 years of follow-up compared to 5% stable BMI.

Another possible reason for the discrepancy between this finding in relation to the mortality risk associated with weight gain and previous studies may arise from insufficient power. It is also possible that obesity and body weight and the risks conferred by excess body mass may vary across different ethnic and racial groups [40]. Moreover, the association between weight gain and mortality risk has been reported to change with age. For example, Chen et al. [41] reported that weight gain from young to middle age was associated with an increased risk of mortality but not for older adults. Similarly, a meta-analysis by Wang [11] showed that the association between obesity and mortality attenuated with increasing age. Collectively, these findings suggest that the mortality risk of weight gain in older adults is not the same as that seen in younger adults, and this may be due to weight generally increasing over the life span [42,43], and those who gain weight at younger ages might have developed some tolerance to that weight gain; therefore, there may be a decreased effect of weight gain on health/survival. Alternatively, that excess weight in later life is mostly due to increased fat mass rather than increasing lean body mass [44], which may provide energy and prevent lean tissue wasting. Therefore, weight gain may be less risky than weight loss in later life.

There are limitations to this study that should be acknowledged. The information on whether the weight loss was intentional or unintentional was not available, and these causes are likely to have different associations with mortality. The issue of reverse causation is acknowledged, unintentional weight loss in later life may reflect an undiagnosed disease or undernutrition linked to mortality. However, in around a quarter of those suffering from weight loss in older age, there is no identifiable cause [45]. In contrast, intentional weight loss may be due to changes in health-related behaviours or decreased energy intake, or increased energy expenditure, which may be beneficial for overall health. A recent meta-analysis reported that studies that were not adjusted for physical activity had a higher risk of mortality in the weight loss group but not in the weight gain group when compared to those that adjusted for physical activity in their analysis [21]. Therefore, physical activity has been identified as a possible confounder of the weight change mortality association. In the current study, the data on physical activity were not available, and thus could not be adjusted for as confounder. Polypharmacy is another factor that may impact both weight and mortality risk [46] but this was not able to be accounted for in these analyses. Changes in waist circumference have been shown to have a strong association with mortality risk in older adults, and it is thought that waist circumference may be a better measurement of risk conferred by adiposity in older adults than weight [14,47,48]. Waist circumference was only available at baseline and therefore change could not be assessed.

It should be noted that models in this study were adjusted for baseline BMI and waist circumference. Another limitation is that the number of underweight participants was small, and data were not available to examine undernutrition specifically. Limitations in the number of fatal events may have impacted statistical power. Another limitation of this study is that weight fluctuation was not measured. As the study used only two time points to assess weight change over a 4-year period, participants who might experience weight fluctuations were not captured, which has been reported to be associated with mortality risk [21]. Finally, participants in ESPRIT were community-dwelling at recruitment, which precludes generalisation of the findings to more frail or non-community dwelling individuals.

This study had a number of key strengths, including that it was a well-characterised, population-based, prospective study with a long (17 year) follow-up and which collected a large amount of risk factor and comorbidity data, enabling extensive covariate adjustment. An additional strength was the validity of the mortality status of the participants, which was obtained using death registries and medical records. The present study also used objectively measured weight at both baseline and follow-up rather than self-reported weight, which a recent systematic review identified as being a significant contributor to heterogeneity [21].

## 5. Conclusions

In this study of community-dwelling adults aged ≥65 years at baseline, weight loss was associated with an increased risk of all-cause and CVD mortality, but no association between weight loss was observed for cancer mortality. Weight loss appeared to be a more serious risk factor for mortality than weight gain in later life. From a public health perspective, these findings highlight the importance of individuals managing weight loss in older adults, rather than weight gain. These findings also highlight the importance of healthcare providers in monitoring the weight change of older adults, as individuals who are losing weight should be offered support strategies to healthily maintain a stable weight. Supportive strategies should include monitoring both daily activity (energy out) and nutritional intake (energy in). Future research is needed to determine whether there are different risks of mortality associated with intentional or unintentional weight loss in later life.

## Figures and Tables

**Table 1:** Baseline characteristics of the participants according to weight change.

Characteristic	Weight Loss	Weight Stable	Weight Gain	*p*-Value
Total *n* (%)	271 (16.3%)	1196 (71.9%)	197 (11.8%)	
Age, years mean (±SD)	74.6 (±5.9)	72.4 (±5.2)	72.3 (±5.3)	0.01
Gender *n* (%)				
Women	167 (61.6%)	686 (57.4%)	132 (67.0%)	0.03
Men	104 (38.4%)	510 (42.6%)	65 (33.0%)	
Weight, kg mean (±SD)	67.8 (±12.9)	67.7 (±12.0)	64.4 (±10.4)	0.007
BMI ^1^, kg/m^2^ mean (±SD)	25.6 (±3.9)	25.0 (±3.5)	24.3 (±3.3)	0.01
BMI group (kg/m^2^) *n* (%)				
Underweight < 18.5	5 (1.9%)	24 (2.0%)	6 (3.1%)	0.01
Normal weight 18.5 to 24.9	120 (44.4%)	641 (53.8%)	118 (60.2%)	
Overweight 25 to 29.9	114 (42.2%)	432 (36.2%)	62 (31.6%)	
Obese ≥ 30	31 (11.5%)	95 (8.0%)	10 (5.1%)	
Waist circumference, mean (±SD)	89.8 (±11.6)	88.3 (±11.8)	87.1 (±11.6)	0.88
Marital status *n* (%)				
Married	167 (61.6%)	810 (67.8%)	131 (66.5%)	0.15
divorced/separated, widow, single	104 (38.4%)	358 (32.2%)	66 (33.5%)	
Smoking status *n* (%)				
Non-smoker	167 (62.6%)	692 (57.9%)	114 (57.9%)	0.52
Past/current smoker	104 (38.4%)	504 (42.1%)	83(42.1%)	
Alcohol consumption *n* (%)				
Non/past alcohol drinker	57 (21.1%)	158 (13.2%)	37 (18.9%)	0.002
Alcohol drinker	213 (78.9%)	1035 (86.8%)	159 (81.1%)	
Education *n* (%)				
Not Completed at least secondary school	176 (65.9%)	766 (64.1%)	136 (69.1%)	0.39
Completed at least secondary school	93 (35.1%)	430 (35.9%)	61 (30.9%)	
Diabetes *n* (%)	38 (14.2%)	96 (8.1%)	14 (7.2%)	0.004
Recent cancer *n* (%)	6 (2.2%)	32 (2.7%)	4 (2.1%)	0.81
Current depression ^2^ *n* (%)	88 (32.7%)	307 (25.9%)	69 (35.6%)	0.004

Weight change: weight loss (≥5%), weight stable (±<5%) and weight gain (≥5%), *n* = number of observations; SD = standard deviation; *p*-values are from ANOVA (continuous variables); chi-square tests (categorical variables). ^1^ BMI: body mass index; (BMI categories): underweight (<18.5), normal (18.5–25), overweight (25–30) and obese (≥30). Diabetes = self-report of diabetes or fasting glycaemia 7.0 mmol/L or reported treatment. ^2^ Current depression = high depressive symptoms (CESD) or current major depressive disorder (MDD).

**Table 2:** The association between weight change and all-cause mortality in older adults aged 65 years or over.

	*n*/*n* Events (%)	Model 1		Model 2		Model 3	
Weight change		HR (95% CI)	*p*-Value	HR (95% CI)	*p*-Value	HR (95% CI)	*p*-Value
Weight loss	271/129 (22.8)	1.27 (1.03–1.56)	0.02	1.28 (1.05–1.57)	0.02	1.24 (1.00–1.56)	0.046
Weight stable	1196/378 (66.9)	1.00		1.00		1.00	
Weight gain	197/58 (10.3)	1.04 (0.79–1.37)	0.76	1.02 (0.77–1.35)	0.83	1.05 (0.76–1.45)	0.74

*n* = number of observations; HR = hazard ratio; CI = confidence interval. Weight change: weight loss (≥5%), weight stable (±<5%) and weight gain (≥5%). Model 1: adjusted for sex (age as time scale in the model). Model 2: adjusted for sex (age as time scale in the model) marital status, education level, smoking status and alcohol consumption. Model 3: adjusted for sex (age as time scale in the model) marital status, education level, smoking status and alcohol depression, diabetes, recent cancer and baseline BMI and waist circumference.

**Table 3:** The association between weight change and CVD mortality in older adults aged 65 years or over (*n* = 1664).

	*n*/*n* Events (%)	Model 1		Model 2		Model 3	
Weight change		HR (95% CI)	*p*-Value	HR (95% CI)	*p*-Value	HR (95% CI)	*p*-Value
Weight loss	271/64 (26.7)	1.52 (1.13–2.04)	0.005	1.51 (1.12–2.05)	0.007	1.53 (1.10–2.14)	0.01
Weight stable	1196/153 (63.5)	1.00		1.00		1.00	
Weight gain	197/24 (11.8)	1.06 (0.68–1.63)	0.79	1.06 (0.70–1.64)	0.77	0.97 (0.57–1.65)	0.93

*n* = number of observations; HR = hazard ratio; CI = confidence interval. Weight change: weight loss (≥5%), weight stable (±<5%) and weight gain (≥5%). Model 1: adjusted for sex (age as time scale in the model). Model 2: adjusted for sex (age as time scale in the model), marital status, education level, smoking status and alcohol consumption. Model 3: adjusted for sex (age as time scale in the model), marital status, education level, smoking status and alcohol consumption depression, diabetes, recent cancer, baseline BMI and waist circumference.

**Table 4:** The association between weight change and cancer mortality in older adults aged 65 years or over.

	*n*/ *n* Events (%)	Model 1		Model 2		Model 3	
Weight change		HR (95% CI)	*p*-Value	HR (95% CI)	*p*-Value	HR (95% CI)	*p*-Value
Weight loss	271/22 (15.4)	0.93 (0.58–1.47)	0.76	0.95 (0.60–1.51)	0.84	0.83 (0.50–1.39)	0.49
Weight stable	1196/103 (71.9)	1.00		1.00		1.00	
Weight gain	197/18 (12,6)	1.17 (0.71–1.94)	0.53	1.17 (0.71–1.94)	0.52	1.15 (0.65–2.04)	0.62

*n* = number of observations; HR = hazard ratio; CI = confidence interval. Weight change: weight loss (≥5%), weight stable (±<5%) and weight gain (≥5%). Model 1: adjusted for sex (age as time scale in the model). Model 2: adjusted for sex (age as time scale in the model) marital status, education level, smoking status and alcohol consumption. Model 3: adjusted for sex (age as time scale in the model) marital status, education level, smoking status and alcohol consumption depression, diabetes, baseline BMI and waist circumference.

## Data Availability

Any requests for data can be sent to the corresponding author.

## Supplementary Materials

The following supporting information can be downloaded at: https://www.mdpi.com/article/10.3390/nu14142983/s1, Figure S1: Flow diagram of study participants included in this analysis; Table S1: Characteristics of ESPRIT participants who were missing at follow-up and had missing weight variable compared with not missing; Table S2: The association between ±5 kg weight change and all-cause mortality; Table S3: The association between ±5 kg weight change and CVD mortality; Table S4: The association between ±5 kg weight change and cancer mortality; Table S5: The association between weight change and all-cause mortality in ESPRIT participants (*n* = 1622, Events = 546), after excluding participants with recent cancer at baseline; Table S6: The association between weight change and all-cause mortality in ESPRIT participants after excluding participants with more than 20 % weight change; Table S7: Hazard ratio (HR) from competing risks survival analysis for causes of deaths other than CVD deaths; Table S8: Hazard ratio (HR) from competing risks survival analysis for causes of deaths other than cancer deaths; Table S9: The association between weight change and all-cause mortality in ESPRIT participants using the time in the study as time scale; Table S10: The association between weight change and all-cause mortality in ESPRIT participants using the age at 4 years follow-up, instead of age at baseline, to generate time scale.
