# Supplementary material for: Objectively Assessed Weight Change and All-Cause Mortality among Community-Dwelling Older People
Source: Nutrients. 2022 Jul 21;14(14):2983. doi: 10.3390/nu14142983 (PMC9320907; doi:10.3390/nu14142983)
Supplement: Supplementary file 1 [file nutrients-14-02983-s001.zip › nutrients-1806873-supplementary.pdf]

nutrients

MDPI

Article

Objectively Assessed Weight Change and All-Cause Mortality among Community-Dwelling Older People

Tagrid Alharbi [1], Joanne Ryan [1], Rosanne Freak-Poli [1,2], Danijela Gasevic [1,3], Jacqueline Scali [4], Karen Ritchie [4,5], Marie-Laure Ancelin [4] and Alice J. Owen [1,*]

1 School of Public Health and Preventive Medicine, Monash University, 553 St. Kilda Rd, Melbourne, VIC 3004, Australia; tagrid.alharbi@monash.edu (T.A.); joanne.ryan@monash.edu (J.R.); rosanne.freak-poli@monash.edu (R.F.-P.); danijela.gasevic@monash.edu (D.G.)
2 Department of Epidemiology, Erasmus Medical Centre, 3015 GD Rotterdam, The Netherlands
3 Usher Institute, University of Edinburgh, Teviot Place, Edinburgh EH8 9AG, UK
4 INM, Université de Montpellier, INSERM, 34000 Montpellier, France; jacqueline.scali@inserm.fr (J.S.); karen.ritchie@inserm.fr (K.R.); marie-laure.ancelin@inserm.fr (M.-L.A.)
5 Centre for Clinical Brain Sciences, University of Edinburgh, Edinburgh EH16 4SB, UK
* Correspondence: alice.owen@monash.edu; Tel.: +61-3-9903-0416

Citation: Alharbi, T.; Ryan, J.; Freak-Poli, R.; Gasevic, D.; Scali, J.; Ritchie, K.; Ancelin, M.-L.; Owen, A.J. Objectively Assessed Weight Change and All-Cause Mortality among Community-Dwelling Older People. *Nutrients* **2022**, *14*, 2983. https://doi.org/10.3390/nu14142983

Academic Editor: Hirofumi Tanaka

Received: 23 June 2022
Accepted: 18 July 2022
Published: 21 July 2022

Publisher's Note: MDPI stays neutral with regard to jurisdictional claims in published maps and institutional affiliations.

Copyright: © 2022 by the authors. Licensee MDPI, Basel, Switzerland. This article is an open access article distributed under the terms and conditions of the Creative Commons Attribution (CC BY) license (https://creativecommons.org/licenses/by/4.0/).

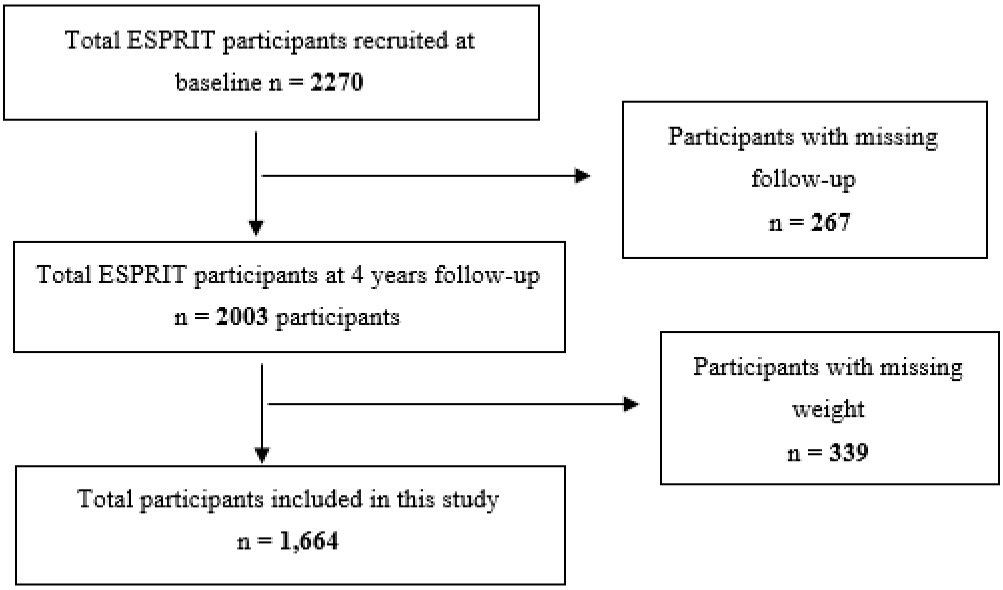

Figure S1. Flow diagram of study participants included in this analysis.

Table S1. Characteristics of ESPRIT participants who were missing at follow-up and had missing weight variable compared with not missing.

	Missing	Not Missing	*p*-Value
**Total *n* (%)**	606 (26.7%)	1664 (73.3%)	
Age, years mean (±SD)	74.9 (±6.2)	72.7 (±5.3)	<0.001
Sex *n* (%)			
Women	328 (55.1%)	985 (59.2%)	0.08
Men	267 (44.8%)	679 (40.8%)	
Weight, kg mean (±SD)	67.3 (±12.0)	67.3 (±12.8)	0.12
BMI [1], kg/m2 mean (±SD)	25.2 (±4.0)	25.0 (±3.5)	0.07
Marital status *n* (%)			
Married	167 (61.6%)	810 (67.8%)	0.15
divorced/separated, widow, single	104 (38.4%)	358 (32.2%)	
Smoking status *n* (%)			
Non- smoker	257(43.3%)	691 (41.5%)	0.46
past/current smoker	337 (56.7%)	973 (58.5%)	
Alcohol consumption *n* (%)			
Non/past alcohol drinker	130 (21.9%)	252 (15.2%)	<0.001
Alcohol drinker	464 (78.1%)	1407 (84.8%)	
Education *n* (%)			
Not Completed at least secondary school	434 (73.2%)	1078 (64.7%)	<0.001
Completed at least secondary school	159 (26.8%)	568 (35.2%)	
Diabetes *n* (%)			
No	503 (87.8%)	1501 (91.1%)	0.02
Yes	70 (12.2%)	148 (9.0%)	
Recent cancer *n* (%)			
No	586 (96.7%)	1622 (97.5%)	0.31
Yes	20 (3.3%)	42 (2.5%)	
Current depression [2] *n* (%)			
No	357 (61.1%)	1186 (74.8%)	<0.001

Yes		227 (38.8%)	464 (28.1%)

[1] BMI: body mass index; (BMI categories): underweight (<18.5), normal (18.5–25), overweight (25–30) and obese (≥30). Diabetes = self-report of diabetes or fasting glycaemia 7.0 mmol/L or reported treatment. [2] Current depression = high depressive symptoms (CESD) or current major depressive disorder (MDD).

Table S2. The association between ±5 kg weight change and all-cause mortality (*n* = 1664).

	***n*/*n* Events (%)**	**Model 1**		**Model 2**		**Model 3**	
**Weight change**		**HR (95% CI)**	***p*-Value**	**HR (95% CI)**	***p*-Value**	**HR (95% CI)**	***p*-Value**
Weight loss	176/94 (16.6)	1.77 (1.41–2.21)	<0.001	1.78(1.42–2.24)	<0.001	1.70 (1.35–2.18)	<0.001
Weight stable	1370/435 (76.9)	1.00		1.00		1.00	
Weight gain	118/36 (6.4)	1.10 (0.78–1.55)	0.56	1.07 (0.76–1.52)	0.69	1.10 (0.74–1.64)	0.62

n = number of observations; HR = hazard ratio; CI = confidence interval. Weight change: weight loss (≥5 kg), weight stable (±<5 kg) and weight gain (≥5 kg). Model 1: adjusted for sex (age as time scale in the model). Model 2: adjusted for sex (age as time scale in the model) marital status, education level, smoking status and alcohol consumption. Model 3: adjusted for sex (age as time scale in the model) marital status, education level, smoking status and alcohol consumption depression, diabetes, baseline BMI and waist circumference**.**

Table S3. The association between ±5 kg weight change and CVD mortality (*n* = 1664).

	***n*/*n* Events (%)**	**Model 1**		**Model 2**		**Model 3**	
**Weight change**		**HR (95% CI)**	***p*-Value**	**HR (95% CI)**	***p*-Value**	**HR (95% CI)**	***p*-Value**
Weight loss	176/45 (18.7)	2.06 (1.48–2.86)	<0.001	2.03 (1.44–2.85)	<0.001	2.04 (1.42–2.95)	<0.001
Weight stable	1370/180 (74.7)	1.00		1.00		1.00	
Weight gain	118/16 (6.6)	1.19 (0.71–1.99)	0.69	1.07 (0.71–1.98)	0.51	1.15 (0.61–2.12)	0.66

n = number of observations; HR = hazard ratio; CI = confidence interval. Weight change: weight loss (≥5 kg), weight stable (±<5%) and weight gain (≥5 kg). Model 1: adjusted for sex (age as time scale in the model). Model 2: adjusted for sex (age as time scale in the model) marital status, education level, smoking status and alcohol consumption. Model 3: adjusted for sex (age as time scale in the model) marital status, education level, smoking status and alcohol consumption depression, diabetes, baseline BMI and waist circumference**.**

Table S4. The association between ±5 kg weight change and cancer mortality (*n* = 1664).

	***n*/*n* Events (%)**	**Model 1**		**Model 2**		**Model 3**	
Weight change		**HR (95% CI)**	***p*-Value**	**HR (95% CI)**	***p*-Value**	**HR (95% CI)**	***p*-Value**
Weight loss	176/16 (11.2)	1.11 (0.66–1.88)	0.68	1.13 (0.67–1.91)	0.4	1.07 (0.61–1.86)	0.80
Weight stable	1370/116 (81.1)	1.00		1.00		1.00	
Weight gain	118/11 (7.7)	1.17 (0.63–2.18)	0.60	1.17 (0.62–2.17)	0.62	1.19 (0.60–2.38)	0.60

n = number of observations; HR = hazard ratio; CI = confidence interval. Weight change: weight loss (≥5 kg), weight stable (±<5 kg) and weight gain (≥5 kg). Model 1: adjusted for sex (age as time scale in the model). Model 2: adjusted for sex (age as time scale in the model) marital status, education level, smoking status and alcohol consumption. Model 3: adjusted for sex (age as time scale in the model) marital status, education level, smoking status and alcohol consumption depression, diabetes, baseline BMI and waist circumference**.**

Table S5. The association between weight change and all-cause mortality in ESPRIT participants (*n* = 1622, Events = 546), after excluding participants with recent cancer at baseline.

**.**		**Model 1**		**Model 2**		**Model 3**	
Weight change	***n*/Events**	**HR (95% CI)**	***p*-Value**	**HR (95% CI)**	***p*-Value**	**HR (95% CI)**	***p*-Value**
Weight loss	265/128	1.31 (1.03–1.61)	0.008	1.33 (1.08–1.63)	0.007	1.30 (1.04–1.63)	0.02
Weight stable	1164/362	1.00		1.00		1.00	
Weight gain	193/56	1.04 (0.79–1.37)	0.77	1.02 (0.77–1.36)	0.87	1.03 (0.74–1.43)	0.85

n = number of observations; HR = hazard ratio; CI = confidence interval. Weight change: weight loss (≥5%), weight stable (±<5%) and weight gain (≥5%). Model 1: adjusted for sex (age as time scale in the model). Model 2: adjusted for sex (age as time scale in the model) marital status, education level, smoking status and alcohol consumption. Model 3: adjusted for sex (age as time scale in the model) marital status, education level, smoking status and alcohol consumption depression, diabetes, baseline BMI and waist circumference.

Table S6. The association between weight change and all-cause mortality in ESPRIT participants after excluding participants with more than 20% weight change.

	n/Events	Model 1		Model 2		Model 3	
**Weight change**		**HR (95% CI)**	***p*-Value**	**HR (95% CI)**	***p*-Value**	**HR (95% CI)**	***p*-Value**
Weight loss	260/123	1.23 (1.00–1.51)	0.04	1.24 (1.00–1.53)	0.04	1.20 (0.96–1.52)	0.11
Weight stable	1196/378	1.00		1.00		1.00	
Weight gain	191/57	1.06 (0.81–1.40)	0.66	1.05 (0.79–1.39)	0.74	1.07 (0.78–1.50)	0.65

n = number of observations; HR = hazard ratio; CI = confidence interval. Weight change: weight loss (≥5%), weight stable (±<5%) and weight gain (≥5%). Model 1: adjusted for sex (age as time scale in the model). Model 2: adjusted for sex (age as time scale in the model) marital status, education level, smoking status and alcohol consumption. Model 3: adjusted for sex (age as time scale in the model) marital status, education level, smoking status and alcohol consumption depression, diabetes, baseline BMI and waist circumference.

Table S7. Hazard ratio (HR) from competing risks survival analysis for causes of deaths other than CVD deaths (*n* = 1664).

Weight Change	HR (95% CI)	*p*-Value
Weight loss	1.58 (1.17–2.12)	0.003
Weight stable	1.00	
Weight gain	0.91 (0.57–1.45)	0.69

Table S8. Hazard ratio (HR) from competing risks survival analysis for causes of deaths other than cancer deaths (*n* = 1664).

Weight Change	HR (95% CI)	*p*-Value
Weight loss	0.79 (0.50–1.25)	0.32
Weight stable	1.00	
Weight gain	1.08 (0.65–1.79)	0.75

Table S9. The association between weight change and all-cause mortality in ESPRIT participants using the time in the study as time scale.

Weight Change	Model 1		Model 2		Model 3	
	**HR (95% CI)**	***p*-Value**	**HR (95% CI)**	***p*-Value**	**HR (95% CI)**	***p*-Value**
Weight loss	1.35 (1.10–1.65)	0.004	1.36 (1.11–1.68)	0.003	1.28 (1.02–1.60)	0.03
Weight stable	1.00		1.00		1.00	
Weight gain	0.95 (0.72–1.25)	0.71	0.91 (0.69–1.21)	0.54	0.92 (0.66–1.27)	0.62

Weight change: weight loss (≥5%), weight stable (±<5%), and weight gain (≥5%). Model 1: adjusted for sex and age. Model 2: adjusted for sex, age, marital status, education level, smoking status and alcohol consumption. Model 3: adjusted for sex, age marital status, education level, smoking status, alcohol consumption, depression, diabetes, recent cancer, baseline BMI and waist circumference.

Table S10. The association between weight change and all-cause mortality in ESPRIT participants using the age at 4 years follow-up, instead of age at baseline, to generate time scale.

Weight Change	Model 1		Model 2		Model 3	
	**HR (95% CI)**	***p*-Value**	**HR (95% CI)**	***p*-Value**	**HR (95% CI)**	***p*-Value**

Weight loss	1.22 (1.00–1.48)	0.05	1.23 (1.00–1.51)	0.05	1.20 (0.96–1.50)	0.10
Weight stable	1.00		1.00		1.00	
Weight gain	1.04 (0.78–1.37)	0.80	1.03 (0.77–1.35)	0.85	1.07 (0.78–1.48)	0.64

Weight change: weight loss (≥5%), weight stable (±<5%) and weight gain (≥5%). Model 1: adjusted for sex (age at 4 years follow-up used to generate time scale in the model). Model 2: adjusted for sex (age at 4 years follow-up used to generate time scale in the model) marital status, education level, smoking status and alcohol consumption. Model 3: adjusted for sex (age at 4 years follow-up used to generate time scale in the model) marital status, education level, smoking status, alcohol consumption, depression, diabetes, recent cancer, baseline BMI and waist circumference.
